# Subcellular localization of ammonium transporters in *Dictyostelium discoideum*

**DOI:** 10.1186/1471-2121-9-71

**Published:** 2008-12-24

**Authors:** Janet H Kirsten, Yanhua Xiong, Carter T Davis, Charles K Singleton

**Affiliations:** 1 Department of Biological Sciences, Vanderbilt University, VU Station B 351634, Nashville TN 37235-1634, USA; 2 LSU School of Medicine – New Orleans, 2020 Gravier Street, New Orleans, LA 70112, USA

## Abstract

**Background:**

With the exception of vertebrates, most organisms have plasma membrane associated ammonium transporters which primarily serve to import a source of nitrogen for nutritional purposes. *Dictyostelium discoideum *has three ammonium transporters, Amts A, B and C. Our present work used fluorescent fusion proteins to determine the cellular localization of the Amts and tested the hypothesis that the transporters mediate removal of ammonia generated endogenously from the elevated protein catabolism common to many protists.

**Results:**

Using RFP and YFP fusion constructs driven by the actin 15 promoter, we found that the three ammonium transporters were localized on the plasma membrane and on the membranes of subcellular organelles. AmtA and AmtB were localized on the membranes of endolysosomes and phagosomes, with AmtB further localized on the membranes of contractile vacuoles. AmtC also was localized on subcellular organelles when it was stabilized by coexpression with either the AmtA or AmtB fusion transporter. The three ammonium transporters exported ammonia linearly with regard to time during the first 18 hours of the developmental program as revealed by reduced export in the null strains. The fluorescently tagged transporters rescued export when expressed in the null strains, and thus they were functional transporters.

**Conclusion:**

Unlike ammonium transporters in most organisms, which import NH_3_/NH_4_^+ ^as a nitrogen source, those of *Dictyostelium *export ammonia/ammonium as a waste product from extensive catabolism of exogenously derived and endogenous proteins. Localization on proteolytic organelles and on the neutral contractile vacuole suggests that *Dictyostelium *ammonium transporters may have unique subcellular functions and play a role in the maintenance of intracellular ammonium distribution. A lack of correlation between the null strain phenotypes and ammonia excretion properties of the ammonium transporters suggests that it is not the excretion function that is important for coupling ammonia levels to the slug versus culmination choice, but rather a sensor and/or signaling function of these proteins that is important.

## Background

The family of ammonium transport proteins (Amt/MEP/Rh) are ubiquitous in all three domains of life [1] and serve to function both in nitrogen uptake for nutritional purposes and ammonia (NH_3_/NH_4_^+ ^without distinction) excretion for waste removal. In plants, bacteria and fungi the most common function of the Amt/MEP proteins is to import ammonia, a primary source of nitrogen, and in some cases the transporters are only activated under conditions of nitrogen starvation [2-6]. The mammalian Rh proteins have been demonstrated to transport ammonia when expressed in yeast or *Xenopus *oocytes, but controversy exists over whether that is their function or a remnant of their evolution from Amts in the distant past [7]. Most recent evidence supports that the primary role of Rh proteins is to translocate CO_2_/HCO_3_[8]. Amts are represented in archaea and invertebrates, are ubiquitous in eubacteria, fungi and plants, and are absent in vertebrates, while Rh proteins are rare in prokaryotes, absent in plants and fungi, scattered in non-fungi microbial eukarya and ubiquitous in animals, especially vertebrates [7]. Amt and Rh proteins coexist only in some microbial eukarya and invertebrates. *Dictyostelium discoideum *is one of these species, having three Amts (AmtA, AmtB, and AmtC) and two Rh proteins (RhgA, RhgB).

Debate exists over whether Amt/MEPs transport NH_3_, NH_4_^+ ^or a combination thereof, and whether they are active transporters or gas channels. In yeast and bacteria, reversible transport of NH_3 _by Amts has been demonstrated [9,10]. While consensus is accumulating around an NH_3 _gas channel in most species, the evidence of active transport of NH_4_^+ ^is strong in plants and remains to be resolved [11]. The crystal structures for *Escherichia coli *AmtB [12,13] and *Archaeoglobus fulgidus *Amt-1 [14] have been elucidated, and the findings lend strong support to Amts functioning as NH_3 _gas channels. The structural studies show that Amts contain 11 transmembrane helices (contrasted with 12 for Rh) with pseudo-twofold symmetry and that their functional state is as trimeric oligomers. Although Amts are believed to naturally form homotrimers, evidence exists that Amts are capable of heteromerization [15] and that some Rh proteins are heteromeric complexes [16], though recently the latter assertion has been challenged [17].

Amts are believed to have several functions in *Dictyostelium *based on its feeding behavior and on its reliance on ammonia production and signaling during development, particularly during the transitional period between slug migration and culmination when final differentiation takes place to form a mature stalk and spores. Ammonia is produced throughout growth and development via oxidative degradation of exogenously obtained or endogenous proteins [18-20] to provide a source of carbon and energy. As a member of the Amoebozoa, *Dictyostelium *is an avid phagocyte that engulfs its food whole, extracting the nutrients within proteolytic organelles rather than differentially importing specific nutrients from the environment, as do plants, bacteria and fungi. Amoebae also live in fresh water environments and lack cell walls, causing them to rely on a contractile vacuolar system to maintain osmotic pressure and ionic homeostasis by expelling excess water [21]. As a result of these physiological processes, the role of Amts in *Dictyostelium *probably contrasts with that of other organisms in that export of excess ammonia produced from protein catabolism is likely to be a primary function.

In its native habitat in the leaf litter of temperate forests, *Dictyostelium *grows as independent amoebae feeding on bacteria and small microbial eukarya. When food stores are depleted, the amoebae coalesce to form multicellular structures that progress through a developmental program characterized by multiple morphological and differentiation events. The program eventually results in a cellulosidic stalk supporting a sorus filled with spores, which are dispersed by wind, water, and passing animals. Midway through the developmental process, a slug is formed that determines from environmental cues whether to migrate or to begin terminal differentiation and culminate. Environmental ammonia concentration appears to be critical for this determination [22,23], with high extracellular ammonia representing a poor environment for spore dispersal and encouraging slug migration and low extracellular ammonia promoting culmination [19]. Two of the Amt proteins, AmtC and AmtA, have been proposed to be ammonia sensors that mediate the slug versus culmination choice via modulation of cyclic AMP dependent protein kinase A through the histidine kinase DhkC phosphorelay [22,24].

In previous work, we investigated the spatial localization of the *Dictyostelium *Amts at the mRNA level using in situ hybridization and found that Amts A, B and C are expressed in the prespore cells that form the posterior 80% of migrating slugs, and in the control center at the tip that is responsible for the slug migration versus culmination decision [22,24,25]. Our present work focuses on determining the cellular localization of the Amts using fluorescent fusion proteins driven by the heterologous actin 15 (Act15) promoter and on testing the hypothesis that the transporters mediate removal of endogenously generated ammonia.

## Results

### Export of Ammonia

Amts in most microorganisms, fungi and plants function to import ammonia in order to obtain nitrogen as a nutrient source. In contrast, *Dictyostelium *cells generate excess ammonia due to the extensive use of protein catabolism during feeding on other microbes or on an enriched broth. The Amt null strains and the parental wild-type strain, Ax4, were used to test if one or more of the Amts function in ammonia release. Excreted ammonia, trapped within buffer soaked supporting pads, as well as intracellular ammonia levels were examined during the developmental program from 12 to 18 hours post-starvation. This is the time period when AmtA and AmtC function in the choice between slug formation versus culmination [22,24].

**Figure 1:**
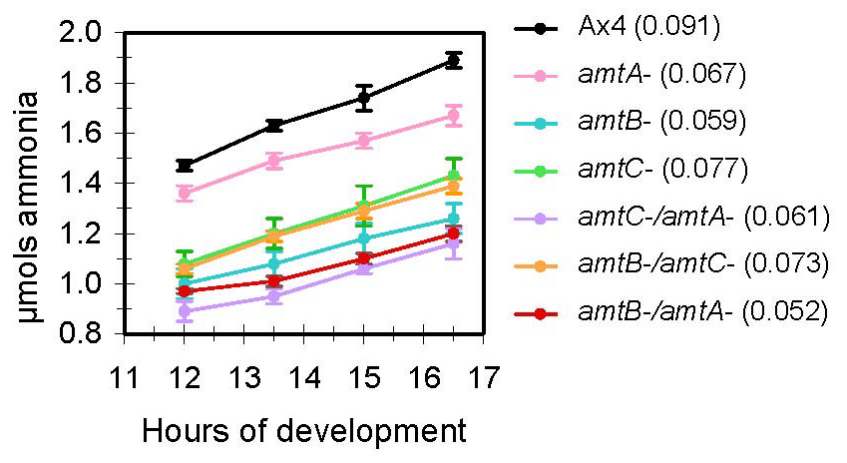
**Excretion of ammonia from developing cells**. Cells from the parental strain (Ax4) and the indicated single and double *amt *null strains were plated for development as described in Methods. Excreted ammonia that collected in the buffer-soaked supporting pads for the indicated times post-starvation was measured using an ammonia specific electrode. Error bars give the standard error from four independent experiments. The slope of each line, representing the micromoles of ammonia released per hour, is given in parentheses following each strain name.

Developing cells excreted ammonia in a linear manner with regard to time after the initiation of development. All strains disrupted in at least one *amt *gene excreted less ammonia and had a reduced rate of ammonia release relative to the parental strain (Figure 1). Ammonia release from the various strains during the first 12 hours of development (not shown) was consistent with that presented in Figure 1, suggesting a relatively constant rate of ammonia excretion from initiation to 18 hours of development that is dependent on the presence or absence of Amts. Similar kinetics of ammonia release were previously observed for the NC4 strain, a wild isolate [19]. While the rates for two of the double null strains were lower than the respective single nulls, this was not the case for *amtB-/amtC-*. No compensatory effect was found at the mRNA level to account for this (not shown).

In contrast to alterations in amounts of ammonia released from the Amt mutant strains, the intracellular levels of ammonia for the single and double null strains were no different from that in the parental strain. In cells growing on bacteria, all strains contained approximately 0.05 micromoles of ammonia per 6 × 10^7 ^cells. By three to five hours post-starvation the intracellular levels in all strains had dropped to about 0.025 micromoles, or half the levels found in growing cells. This level was maintained for at least 18 hours post-starvation in all strains.

**Figure 2:**
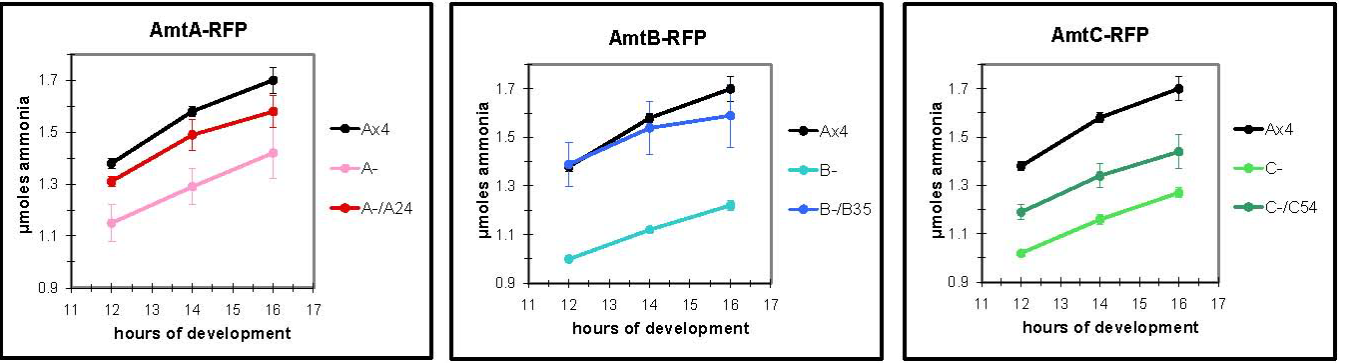
**Comparison of ammonia excretion from the developing *amt *null strains either expressing or not expressing the respective Amt-RFP fusion protein**. Cells from the parental strain (Ax4), the null strains (A-, B-, C-), and the null strains expressing the respective Amt-RFP protein (A-/A24, B-/B35, C-/C54) were plated for development as described in Methods. Excreted ammonia that collected in the buffer-soaked supporting pads for the indicated times post-starvation was measured using an ammonia specific electrode. Error bars give the standard error from three independent experiments.

Using the Act15 promoter, fusion constructs of the Amts with C-terminus YFP and RFP tags were made and transformed into the relevant null strains. To test the functionality of the fusion proteins, excreted ammonia from the transformed null strains was compared to that of the untransformed parental and null strains. For each of the three null strains, ammonia excretion was increased when the strains were transformed with the plasmid encoding the appropriate RFP fusion protein (Figure 2). For the *amtA *and *amtB *null strains, excretion levels were equivalent to or slightly less than that of the parental strain possessing the non-disrupted, endogenous *amt *gene. Expression of AmtC-RFP in *amtC*- cells resulted in ammonia excretion levels that were intermediate to those of the parental and *amtC *null strains. The respective YFP fusions also restored normal ammonia excretion levels when expressed in the *amtA *and *amtB *null strains but not in the *amtC *null (not shown).

### AmtA Cellular Localization

**Figure 3:**
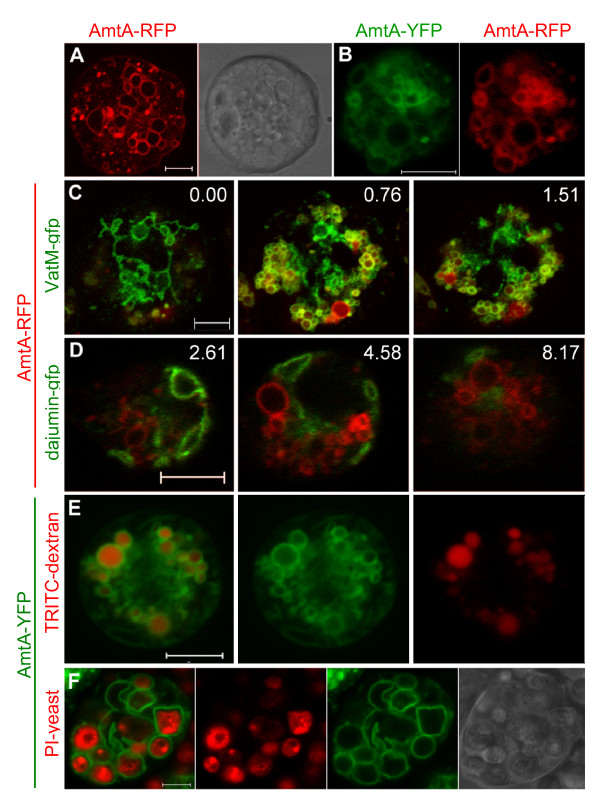
**Localization of AmtA in vegetative cells**. (A) Plasma membrane localization in a typical, growing AmtA-RFP cell with corresponding DIC image. (B) Comparison of AmtA-YFP (left) and AmtA-RFP (right) co-expressed in the same cell. (C) Colocalization (yellow) of AmtA-RFP (red) with contractile vacuoles and endolysosomes (green) labeled with VatM-GFP in Ax2-VatM-GFP cells. Three images from a Z-stack; numbers indicate μm distance above the plane of the first photograph (0.00) in the series. (D) Mutually exclusive localization of AmtA-RFP (red) and contractile vacuoles (green) labeled with Dajumin-GFP in Ax2-Dajumin-GFP cells. Three images from a Z-stack, numbers indicate μm distance above the plane of the first photograph in the series. (E) Localization of AmtA-YFP on endolysosome membranes. AmtA-YFP (green) cells were incubated with TRITC-dextran (red) to identify the lumens of endolysosomes. (F) Localization of AmtA-YFP on phagosome membranes and corresponding DIC image. AmtA-YFP (green) cells were incubated with propidium iodide stained yeast (red) to identify the lumens of phagosomes. All images are confocal with 5 μm scale bars.

The fusion constructs of the three Amts were transformed into Ax4, and the same localization patterns were seen in growing cells of both the wild type and null strains. To our knowledge, for all previous species examined, the Amt/MEP/Rh proteins have been found exclusively on the plasma membrane (see reviews [1,7,26]) with the sole exception being RhgA, one of two Rh homologues in *Dictyostelium *[27]. As expected, AmtA-RFP was localized on the plasma membrane, and surprisingly, more strongly on subcellular organelles (Figure 3A). This also was true for AmtA-YFP, and when the two fusion proteins were co-expressed to confirm their interchangeability, colocalization was essentially 100% (Figure 3B). Because the internal organelle fluorescence levels were so high, resolving them photographically frequently required lowering the exposure time to such an extent that the plasma membrane localization was no longer visible. Expression of YFP and RFP without being fused to an Amt resulted in cytosolic localization for both proteins.

Well defined subcellular membrane and organellar markers exist for *Dictyostelium*. Colocalization of a fluorescently tagged protein expressed by the Act15 promoter with the markers has been used extensively to define subcellular localization [28-31]. To begin defining AmtA subcellular localization, the AmtA-RFP construct was transformed into a VatM-GFP strain. VatM is the *Dictyostelium *homologue to the V_0 _domain of H^+ ^V-ATPases and is responsible for translocation of protons across various membranes. In *Dictyostelium*, VatM is localized strongly on the membranes of the contractile vacuolar system, and 10-fold less densely on the membranes of phagosomes and most endolysosomes (post-lysosomes lose their proton pumps) [32]. Figure 3C shows representative images from three focal planes in a Z stack of a gently flattened, vegetative cell, co-labeled with AmtA-RFP and VatM-GFP. Near the bottom of the cell (0.00 μm), the characteristic contractile vacuole pattern of VatM-GFP was present, and no AmtA-RFP appeared to be colocalized with it. More internally (0.76–1.51 μm), colocalization became apparent with numerous yellow circular membranes, while some VatM-GFP localized alone. One circular organelle at the bottom of Figure 3C was labeled with only AmtA-RFP and may be a late endosome/post-lysosome that has already lost VatM-GFP.

To confirm the absence of AmtA in the contractile vacuolar system, AmtA-RFP was transformed into a strain expressing dajumin-GFP [33], which exclusively labels contractile vacuolar membranes. The images in Figure 3D are from three representative focal planes in a Z-stack showing that AmtA-RFP and dajumin-GFP were mutually exclusive from one another throughout the cell, confirming that AmtA was not localized on contractile vacuolar membranes.

Having excluded the contractile vacuolar system, we expected the colocalized membranes in the VatM-GFP/AmtA-RFP strain to be vesicles in the endolysosomal system. To confirm this, AmtA-YFP cells were incubated with TRITC-dextran for at least one hour to label the lumen of endosomes. The results in Figure 3E indicate that many of the AmtA-YFP labeled organelles were endolysosomes. To determine if AmtA also was localized to phagosomes, AmtA-YFP cells were incubated with heat-killed yeast cells that had been stained with propidium iodide (Figure 3F). The cells were prodigious feeders and seemingly every AmtA-YFP labeled membrane within the feeding cells was recruited to the phagosomes.

### AmtB Cellular Localization

**Figure 4:**
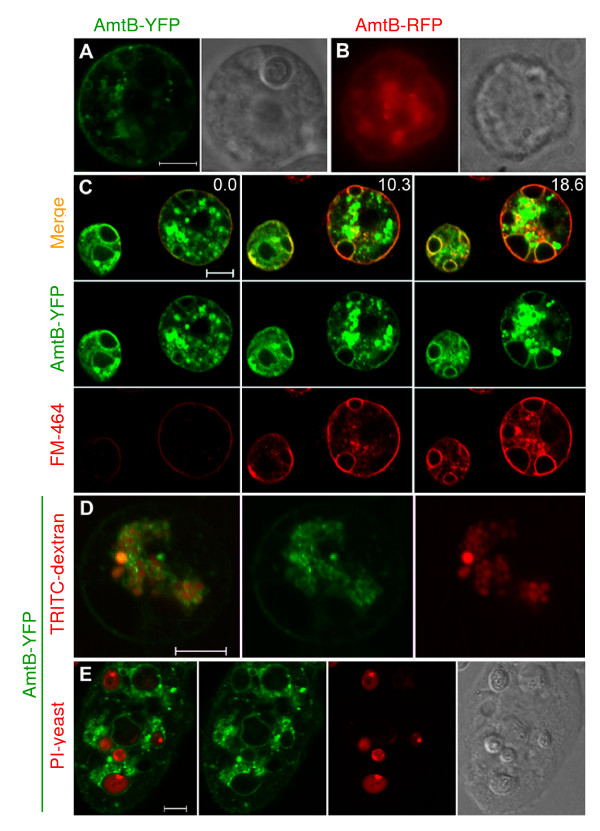
**Localization of AmtB in vegetative cells**. (A) Plasma membrane localization in a typical, growing AmtB-YFP cell with corresponding DIC image. (B) Compound image of plasma membrane localization of a typical, growing AmtB-RFP cell with corresponding phase contrast image. (C) Contractile vacuole membrane colocalization of AmtB-YFP (green) and FM4–64 (red). Three frames are shown from a time series started approximately 1 minute after adding FM4–64 to cells in a well of an 8-chamber cover glass. Images were made every 2 minutes for 10 cycles. Numbers represent number of minutes since the first frame was photographed. (D) Localization of AmtB-YFP on endolysosome membranes. AmtB-YFP (green) cells were incubated with TRITC-dextran (red) to identify the lumens of endolysosomes. (E) Localization of AmtB-YFP on phagosome membranes and corresponding DIC image. *AmtB *null cells expressing AmtB-YFP (green) were incubated with propidium iodide stained yeast (red) to identify the lumens of phagosomes. Images A, C, D, and E are confocal with 5 μm scale bars.

AmtB fused to YFP or RFP was localized on the plasma membrane (Figures 4A and 4B). While AmtB-YFP labeled numerous internal vesicle membranes, AmtB-RFP was less stable, with no recognizable internal organelle membrane localization. Even so, both AmtB fusion strains demonstrated complete recovery of ammonia export levels in our assay, suggesting that plasma membrane localization may be sufficient for that process.

The AmtB-YFP strain was used to study the intracellular localization of AmtB. Because AmtB-YFP and the strains with GFP-labeled organelles both used G418 resistance, determining if AmtB was localized on the contractile vacuole was done with the vital dye FM4–64. Pexophagy studies with yeast have used FM4–64 to delineate vacuolar membranes for 3–9 hours [34] and pulse experiments with *Amoeba proteus *have demonstrated the stability of FM4–64 on contractile vacuoles during contraction cycles for at least 4 hours after removal of the dye [35]. In *Dictyostelium*, FM4–64 initially stains the plasma membrane, moves onto the contractile vacuolar membranes over the course of the next 15–20 minutes, and remains stable on the vacuolar membranes for numerous contraction cycles [36,37]. Figure 4C is three frames from a time series of AmtB-YFP cells stained with FM4–64, with photography commencing approximately one minute after addition of the dye. A strong colocalization signal was present almost immediately on the plasma membrane and colocalization appeared on numerous vacuoles within the cells by the end of the time series. This experiment was repeated in two independently transformed strains of Ax4 and an *amtB*- strain. The pattern was consistent in all of the strains, strongly suggesting that AmtB was localized on the membrane of contractile vacuoles.

To determine if AmtB was localized on endolysosome membranes, endosomes were loaded with TRITC-dextran as described for AmtA. Figure 4D clearly shows numerous TRITC filled vesicles with YFP labeled membranes, confirming the presence of AmtB within the endolysosomal system. Incubating the cells with propidium iodide stained yeast demonstrated that AmtB also was localized on phagosomes (Figure 4E).

### Cell Membrane Orientation

**Figure 5:**
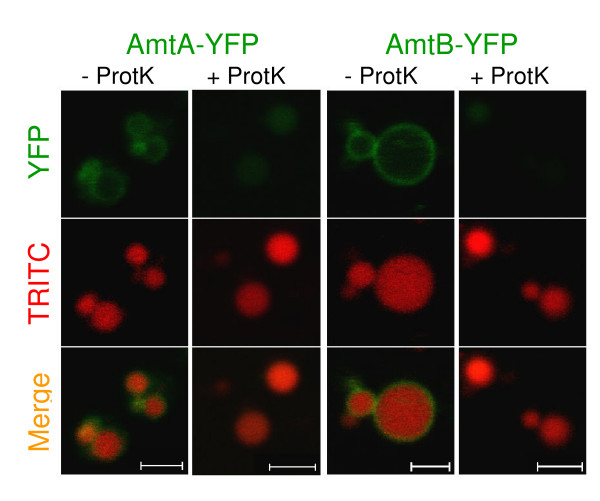
**Amt Topology**. Cytosolic localization of C-terminal YFP tags. The AmtA and AmtB YFP strains (green) were incubated with TRITC-dextran to label endosome lumens (red). After cell lysis, the endosomes were concentrated by centrifugation, and the resuspended pellet was left untreated or treated with proteinase K. Images are confocal with 2 μm scale bars.

Amt/MEP proteins characterized in other species have been determined to have 11 transmembrane helices with the amino terminus exterior to the cell and the carboxy-terminus within the cytosol [12-14]. To examine whether *Dictyostelium *ammonium transporters were oriented similarly, cells expressing the C-terminal tagged transporters were incubated with proteinase K and the cell membrane fluorescence recorded. For both the AmtA and AmtB strains, fluorescence remained on the plasma membrane after protease treatment, in agreement with the expected topology (not shown). To more directly establish topology within the intracellular membranes, endosomes of YFP strains were loaded with TRITC-dextran, concentrated after cell lysis, and treated with or without protease. As shown in Figure 5, protease treatment removed the green rings, while the vesicles remained intact as revealed by the red "balls". This confirms an orientation for the transporters with a cytosolic C-terminal YFP.

### AmtC Localization

**Figure 6:**
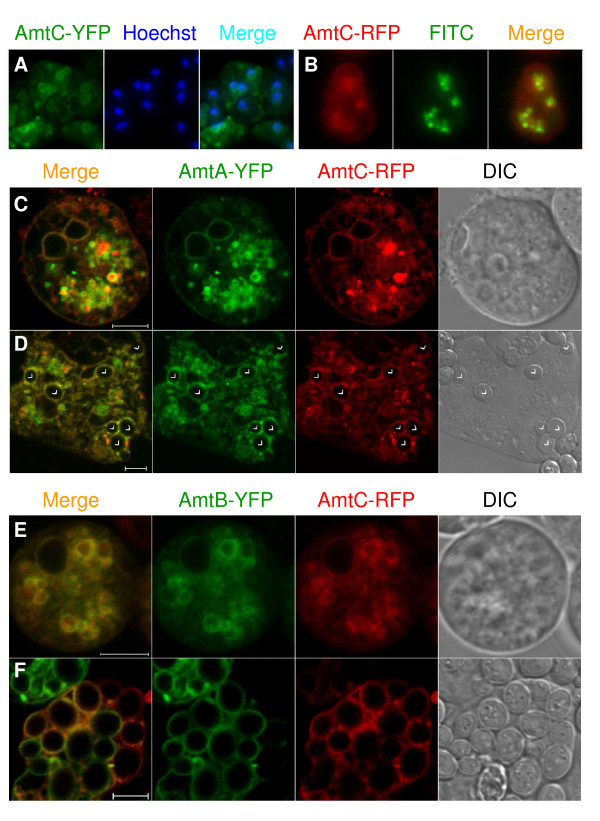
**Localization of AmtC in vegetative cells**. (A) Perinuclear localization of AmtC-YFP. Compound images of AmtC-YFP (left), Hoechst stained nuclei (middle) and merged (right). Cells were fixed briefly in 70% ethanol and washed with DB prior to and after staining with Hoechst. Several cells are shown. (B) Plasma membrane localization of AmtC-RFP. Compound images of AmtC-RFP (left), FITC-dextran loaded endosomes (middle) and merged (right). (C) AmtC-RFP localization on the plasma membrane and some internal organelle membranes when co-expressed with AmtA-YFP in growing cells. (D) Localization of AmtC-RFP on phagosome membranes when co-expressed with AmtA-YFP. Cells were incubated with live yeast (arrowheads) to induce phagocytosis. (E) Apparent complete colocalization of AmtC-RFP with AmtB-YFP in growing cells. (F) Localization of AmtC-RFP on phagosome membranes when co-expressed with AmtB-YFP. Cells were incubated with live yeast to induce phagocytosis. Images C-F are confocal with 5 μm scale bars.

Expression of AmtC-YFP resulted in strong internal organellar membrane labeling, but visualizing the nuclei with Hoechst stain in fixed cells demonstrated that the majority of the rings surrounded nuclei (Figure 6A). This finding suggests that the protein was not successfully exporting from the ER, perhaps due to improper folding or assembly. While it is possible that AmtC normally localizes to the ER, it seemed unlikely given that the ammonia export assay showed that AmtC-YFP had export activity no higher than the *amtC *null strain and that AmtC-RFP had weak plasma membrane localization (Figure 6B). In addition, the ammonia export assay demonstrated that the AmtC-RFP strain had a significant increase in ammonia export relative to the null strain (Figure 2). Even so, most AmtC-RFP fluorescence was within organelles, suggesting significant misfolding or improper assembly in the ER and degradation in endolysosomes, where it colocalized with FITC-dextran (Figure 6B).

The AmtC-RFP strain was transformed with either the AmtA-YFP or the AmtB-YFP fusion construct, hoping at a minimum to obtain a colocalization signal on the plasma membrane. When AmtC-RFP and AmtA-YFP were co-expressed, colocalization was apparent on the plasma membrane, with AmtC-RFP having a consistent and stronger signal than it did when expressed alone. Additionally, AmtC-RFP fluorescence levels on the plasma membrane frequently were stronger than that of AmtA-YFP. Surprisingly, some colocalization of AmtC and AmtA on vesicle membranes also was observed (Figure 6C), although there was considerable variation between cells, as might be expected given the unavoidable variability in plasmid copy numbers of the co-transformed constructs. When the cells were incubated with live yeast, both Amts were observed on phagosome membranes (Figure 6D, arrows) in addition to the membranes of other vesicles.

In comparison to AmtC-RFP/AmtA-YFP, colocalization of AmtC-RFP with AmtB-YFP appeared complete in non-phagocytosing cells (Figure 6E). When cells were incubated with live yeast to induce phagocytosis, there was more variation, with AmtC and AmtB being present either separately or colocalized on phagosome membranes, at times within the same cell (Figure 6F).

The results of co-expression of AmtC with either AmtA or AmtB indicate a stabilization of AmtC relative to when it was expressed alone and successful targeting of AmtC to various organelles, including phagosomes. Stabilization might arise by formation of heterotrimers with the respective co-transformed transporter. However, given the variable relative expression levels during colocalization and the apparent localization of AmtC alone on some membranes during co-expression, AmtC may be stabilized by unknown means that produced independently targeted homotrimers or anomalous heterotrimer formation that resulted in AmtC monomers being targeted to internal organelles where they would not normally be found.

## Discussion

Ammonium transporters in *Dictyostelium *differed from those characterized in other organisms in that they were localized on subcellular organelles, in addition to the plasma membrane. Subcellular localization of *Dictyostelium *Amts is not surprising given the extensive number of roles ammonia has been documented to play in this organism. These range from the general dependence on protein catabolism as a major source of carbon and energy to *Dictyostelium*'s nuanced reliance on ammonia as a signal to monitor both the environment and its own morphological changes and differentiation events during development.

*Dictyostelium *cells feed by phagocytosis and micro- and macro-pinocytosis, resulting in estimates of the turnover of one cell surface equivalent of membrane in as little as 4–10 minutes [38]. Protein degradation and amino acid catabolism of ingested food likely generate large quantities of ammonia within the proteolytic organelles, and nitrogen needs within other organelles and the cytosol necessitate an ability to transport ammonia among these locations. The observed intracellular distribution of the Amts provides a means to maintain appropriate intracellular and organellar distribution of ammonia. The Amts within the plasma membrane and the extracellular release of waste products and undigested materials by the endolysosomal system and phagosomes provide several possible mechanisms for removal of excess ammonia. While *Dictyostelium *is the first species to be identified with Amts on internal organelles, it also is the only member of the Amoebozoa to be examined for Amt localization. We predict that a similar intracellular distribution of Amts will be found within other Amoebozoan species that share feeding mechanisms and environments similar to those of *Dictyostelium*.

The localization of at least one Amt on the contractile vacuole is consistent with the reliance of amoebae on this unique organelle in maintaining osmotic and ionic homeostasis [39]. Protozoa generally live in hypotonic environments and lack cell walls, necessitating an organelle to sequester and expel excess water. This requires a source of expendable and osmotically active ion species to draw water into the vacuole, and it has been suggested that ammonium bicarbonate may be the primary species [36]. In *Dictyostelium*, the non-acidic nature of the contractile vacuole and the presence of AmtB to provide a means of ammonia translocation into the vacuole lend support to this theory. Interestingly, one of the two *Dictyostelium *proteins related to the mammalian Rh proteins has been shown to be localized on the contractile vacuole membrane [27], suggesting a coevolution of function. CO_2_, which is readily hydrated to bicarbonate, is thought to be transported by Rh proteins [7,8], suggesting that RhgA and AmtB may function to provide the osmotic ions in accord with Heuser and Clarke's predictions [36]. Recent work on the tunicate *Ciona intestinalis *has suggested an analogous role for ammonium transporters in maintaining the appropriate density and viscosity of brain fluid necessary for proper brain development in *Ciona *larva [40]. However, in a preliminary experiment in which the transporter mutant strains and the parental strain were treated to a three hour hypotonic shock, no gross morphological differences were found nor was the survivor rate different when the treated cells were plated on bacteria.

Our findings for the strains co-expressing two of the tagged transporters suggest that heteromerization might occur between AmtC and Amts A and B. Rh proteins, a distantly related family of transporters, are thought to have different heteromeric forms possessing different transport characteristics and tissue localization patterns [1,7,41]. LeAmt1;1, and LeAmt1;2, high and low affinity ammonium transporters in tomatoes, are colocalized in root hairs and their co-expression in *Xenopus *oocytes indicated that heteromeric complexes were formed [15]. If heteromers normally form for the *Dictyostelium *Amts, this would produce multiple forms of the transporters, each of which could differ in affinity for ammonium ions, in transport activity, and perhaps in function.

In agreement with previous work [18-20,42], ammonia was excreted linearly during development, with all three of the ammonium transporters contributing and functioning to remove ammonia from developing cells. While the rate of ammonia release was reduced in strains disrupted for any of the transporters, the non-additive reduction in the double null strains indicates complexities not elucidated by these initial characterizations. Our findings for AmtA are consistent with recently published work which also showed a defect in ammonia excretion in an *amtA *null strain [43]. While one means to account for decreased excreted ammonia in the null strains would be a parallel increase in intracellular ammonia content, we found no such increase. In contrast, a small increase was found for *amtA *null cells by Yoshino *et al*. [43]. The different findings may be due to the use of different strains (Ax2 for Yoshino versus our Ax4) or from differences in using cells growing axenically (Yoshino) versus feeding on bacteria (herein). In our analysis, during the first few hours of development the intracellular levels of ammonia decreased in the wild-type strain and similarly in all of the null strains. By three to five hours post-starvation, intracellular ammonia levels for all strains stabilized at roughly half that in growing cells. The observed intracellular ammonia reduction in all of the strains apparently reflects a normally occurring alteration in catabolism in developing versus growing cells. The lack of correlation between intracellular ammonia content and ammonia excretion suggests that in the null strains, homeostatic mechanisms alter the rate of protein catabolism to compensate for loss of one or more Amts in order to maintain appropriate overall internal ammonia levels.

## Conclusion

During development, ammonia excretion likely is a function of the Amt transporters in the cell types that retain them after the onset of starvation, and the excretion assay examines global excretion from the developing, multicellular structures. During the transitional period when a determination is being made to migrate as slugs or to culminate, with high environmental ammonia promoting slugs and low ammonia promoting culmination, we found that all of the *amt *null strains released less ammonia than the wild-type strain. Thus, all of the null strains are surrounded by an environment with less ammonia than that of the wild-type strain, and yet *amtC*- (and the *amtC*/*amtB *double null strain) results in a slugger phenotype [25]. The *amtA *null strain excretes more ammonia than the *amtC- *slugger strain, yet it bypasses the slug stage [24]. These findings appear to support the previously postulated sensor and/or signaling function of these proteins [22,24] in coupling ammonia levels to the slug versus culmination choice.

## Methods

### Strains and Culturing

Strains of *Dictyostelium *were maintained as axenic cultures in HL5 medium [44] in flasks or petri dishes and on SM plates with *Klebsiella pneumoniae *as a bacterial food source [45]. *Dictyostelium discoideum *strain Ax4 was the wild-type strain used in all experiments and was the parental strain for the *amt *null strains. The *amt *null parental strains transformed with fluorescent fusion constructs were blasticidin resistant and were: BS155 (*amtA*-) [24]; BS167 (*amtB*-) (JHK, unpublished); and BS154 (*amtC-*) [25]. The double null strains used were: BS165 (*amtC*-/*amtA*-) [24]; BS169 (*amtB*-/*amtA*-) (YX, unpublished); and BS168 (*amtB*-/*amtC*-) (YX, unpublished). Parental strains with GFP-labeled organelles were G418 resistant and were: Ax2-VatM-GFP (DBS0235537) and Ax2-dajumin-GFP (DBS0236185). These were obtained from the *Dictyostelium *Stock Center [46]. All localization experiments were done on vegetative, exponentially growing cells.

### Fusion Constructs

The coding region of each ammonium transporter was amplified from a cDNA clone (ddv30m16 for *amtA *and ddc19i14 for *amtB*) or reverse transcribed from mRNA (*amtC*) with primers containing in-frame artificial BamHI restriction sites and were ligated into the T-Easy Vector (Promega). The cDNA clones were obtained from the *Dictyostelium *cDNA project [47,48]. After sequencing to obtain clones without mutations, the coding regions were released by restriction digest with BamHI and were ligated into the BamHI sites of pDXA-mcs-YFP, pDXA-YFP-mcs [49], pDXA-GFP2 and pTX-GFP [50]. Positive colonies were identified by PCR and appropriate restriction digests to check orientation. The resulting plasmids contained the *amt *coding regions fused to YFP or GFP at the N or C terminus, as well as a G418 resistance cassette. After introduction into Ax4 cells by electroporation [51,52], only the C-terminal fusion constructs in the pDXA-mcs-YFP backbone fluoresced. This result paralleled earlier work with N- and C-terminal FLAG tag vectors, in which only the C-terminal constructs were successfully stained (JHK/CTD, unpublished), suggesting that alteration to the N-terminus prevents proper protein folding and/or membrane insertion. Additionally, only the pDXA-mcs-YFP vector contained a flexible linker to separate the fluorophore from the transporter, and this may have allowed protein maturation to progress.

Based on this information, a C-terminal fusion vector with hygromycin resistance was constructed using mRFPmars as the fluorophore, pDXA-mcs-RFPmars-Hygro. The coding region for mRFPmars [53] was amplified from 339-3: mRFPmars in pBsrH (ID 1), which was obtained from the *Dictyostelium *Stock Center [46], using the primers RFP-1 (atgcatCA(GGAGGATCA)_3_GGAATGGCATCATCAGAAGATG) and RFP-2 (TAGACATTCAACAGGTGCATAAtctaga). RFP-1 has an artificial NsiI site followed by a Ser-Gly linker prior to the start codon and the first 16 bases of RFPmars. RFP-2 is the last 19 bases of RFPmars, followed by a stop codon and an artificial XbaI restriction site. The amplified product was cloned into the T-Easy vector, verified by sequencing, released with NsiI and XbaI, and ligated into pDXA-3H-Hygro [49] that had been digested with the same restriction enzymes. The coding regions of the ammonium transporters were ligated into pDXA-mcs-RFPmars-Hygro as described above after linearization with BamHI.

All of the C-terminal YFP and RFP fusion constructs were transformed into Ax4 and the respective null strains by electroporation. The RFP fusion constructs additionally were transformed into the strains Ax2-VatM-GFP and Ax2-dajumin-GFP.

### Ammonium Determination

Cells were grown in association with bacteria that were removed by low speed centrifugation as described [45], except that sodium phosphate replaced the potassium phosphate in the standard PDF buffer because the potassium ions interfered with ammonium detection. Cell numbers were determined by direct counting. Within 60 mm Petri dishes, 6 × 10^7 ^cells were plated for development on filters (Millipore HABP04700) atop cellulose pads saturated with 1.9 ml Na-PDF. Dishes were placed within a humidity chamber with overhead light during development. Because the pH of the Na-PDF buffer was 6.5, most ammonia released was retained in the pads as ammonium ions. At appropriate time points, filters were removed from the pads, and 4 ml of 0.1× ISA (0.25 M Mg_2_Acetate, 0.5 M acetic acid), the buffer recommended for use with the ammonium electrode, was added. After gently rotating the dishes for 20 minutes, the pads were removed and the ammonium concentration of the buffer was determined by using an ammonium-specific electrode with a sensitivity and linear range of measurements between 0.01 to 100 mM (Orion 93-18, Thermo Electron Corporation, Beverly, MA). The number of cells and volumes used resulted in ammonium concentrations (0.1–0.5 mM) well within the linear range of the electrode. In some instances, the cells were washed from the filters using Na-PDF, pelleted, and resuspended in 2 ml 0.1× ISA. The cells were lysed with glass beads and vigorous vortexing with completion of lysis confirmed microscopically. Intracellular ammonium levels (in the range of 0.01–0.1 mM) were determined using the ammonium electrode, and the protein concentration of each sample was determined using a Coomassie Plus assay kit (Pierce, Rockford IL). Inclusion of detergent (0.5% NP-40) during lysis did not change the amount of detected intracellular ammonium.

### Protease Experiments

Exponentially growing cells of the AmtA-YFP and AmtB-YFP strains were transferred from HL5 to a low fluorescence axenic medium (LoFlo) [54] and 1 × 10^5 ^cells in 300 μl were placed in the well of an 8-well Lab-tek chambered cover glass (Nunc 155411) and allowed to settle for 30–60 minutes. The cover glass was placed on the microscope stage and after photographing a group of cells with obvious cell membrane localization, 100 μg of proteinase K was added to the well without disturbing the cells. The cells were observed and photographed 10 minutes later to determine if fluorescence remained on the plasma membrane.

For membrane topology determinations of organelles, exponentially growing cells (1 × 10^8^) of AmtA-YFP and AmtB-YFP were incubated with TRITC-dextran (0.4 mg/ml) in 15 ml of 50:50 HL5:LoFlo for 2 hours, pelleted by centrifugation, and washed 3× in ice-cold homogenization buffer (HB) (20 mM HEPES, 0.5 mM EGTA, 25 mM sucrose, pH 7.6). The cells were lysed by suspending in 1 ml of HB, drawing into a syringe, and passing through a stack of two 5.0 μm filters (Millipore SVLP02500) using a Swinnex Filter Holder (Millipore SX0002500). The lysate was centrifuged at 2,000 g for 5 minutes at 4°C to remove nuclei and unlysed cells, and the resulting supernatant was centrifuged at 37,000 g for 10 minutes at 4°C to pellet the remaining cellular organelles, including intact TRITC-dextran loaded endosomes. The pellet was resuspended in 100 μl of ice-cold HB and divided between two tubes, one of which contained 50 μg of proteinase K. After incubating on ice for 10 minutes, the concentrated endosomes were quickly warmed to room temperature, mixed with an equal volume of molten 4% low-melt agarose in HB and quickly compressed between two cover glasses separated by #2 bridges. Microscopic examination commenced immediately.

### Colocalization Studies

In order to determine which organelles the ammonium transporters were localized on, the RFP fusion constructs were transformed into Ax2-VatM-gfp, which is localized on the membranes of the contractile vacuolar system, endolysosomes and phagosomes [32], and into Ax2-dajumin-gfp, which is localized only on contractile vacuole membranes [33]. Colocalization of the ammonium transporters with one another was examined by transforming a second fusion construct into an existing fluorescing strain (Ax4 or relevant null) resulting in co-transformed strains expressing: AmtA-RFP/AmtA-YFP, AmtC-RFP/AmtA-YFP, and AmtC-RFP/AmtB-YFP.

Specific testing of membrane localization in the endolysosomal system was done by loading the endosomes with fluorescent dextrans [55-57]. FITC-dextran (70 kDa; Sigma FD70S) was used in the RFP strains and TRITC-dextran (65–76 kDa; Sigma T1162) in the YFP strains. Briefly, 1 × 10^7 ^cells were washed three times in DB (5 mM Na_2_HPO_4_, 5 mM KH_2_PO_4_, 1 mM CaCl_2_, 2 mM MgCl_2_, pH 6.5.) and resuspended in 1 ml of DB containing 1–2 mg/ml of the fluorescent dextran in a 2 ml microcentrifuge tube. The cells were incubated with rocking in the dark for 1–2 hours. An equal volume of 0.4% Trypan Blue (Sigma T8154) was added to quench any unincorporated fluorescence, and the cells were washed four times in DB prior to depositing 1 × 10^5 ^cells in 300 μl into a well of a chambered cover glass for photography.

FM 4–64 (Molecular Probes) was used to test contractile vacuolar colocalization in the AmtB-YFP strain [36]. Cells were allowed to attach in the well of a chambered cover glass for 30 or more minutes in 250 μl of DB. After placement on the microscope stage, 250 μl of the steryl dye FM4–64 (2 μg/ml) was added to the well, resulting in a 1 μg/ml final concentration. Time-lapse photography commenced almost immediately, with photographs taken every 2 minutes for 10 cycles.

Phagocytosis experiments to test phagosome colocalization were done with YFP strains using heat killed yeast (80°C for 5–10 minutes) that were subsequently non-specifically stained with 5 mM propidium iodide in PBS for 15 minutes [58]. The stained yeast were washed three times in PBS, resuspended in DB, and stored frozen at -20°C. *Dictyostelium *cells (2 × 10^6^) were suspended in 1 ml DB with 1.2 × 10^7 ^thawed yeast cells [59] and were rocked in the dark for 1 hour. Cells (2 × 10^5^) and yeast were allowed to settle on cover slips for a minimum of 30 minutes and were gently flattened with agar overlays [60] immediately prior to photographing. Similar experiments were carried out with strains expressing two Amts using live yeast.

### Microscopy and Image Handling

All images except Figures 4B and 6A and 6B were photographed on a Zeiss LSM510 inverted confocal microscope using a Plan-Apochromatic 63×/1.4 oil DIC or a Plan-Neofluar 100×/1.3 oil DIC lens. Excitation of GFP, YFP and FITC was with the 488 band of the argon laser using a 515–530 or 515–550 nm emission filter. The same 488 nm argon laser was used for FM4–64 but using a 650 nm long-pass emission filter. RFP, TRITC and propidium iodide excitation was with the HeNe1 543 nm laser using a 560 nm long-pass emission filter. Figures 4B and 6A and 6B were photographed on a Nikon Diaphot-TMD inverted compound microscope with an Olympus 100× oil immersion lens using a Q-Imaging Retiga 1300 CCD camera with FITC (Chroma 41001 (Ex. 460–500/BP510–560)), rhodamine (Nikon DM580 (Ex. 510–560/LP590)) and DAPI (Nikon UV2A (Ex. 330–380/LP420)) filters. Confocal images used in the figures were exported from the Zeiss LSM Image Browser, imported into Simple PCI (Hamamatsu Corp.) to assemble exportable panels, and lastly imported into Microsoft Word for figure assembly. Minimal to no alterations were made of the original images beyond cropping as necessary.

## Abbreviations

Amt: ammonium transporter; DB: development buffer; ER: endoplasmic reticulum; FITC: fluorescein isothiocyanate; G/R/YFP: green/red/yellow fluorescent protein; HB: homogenization buffer; MEP: methylammonium permease; PBS: phosphate buffered saline; PI: propidium iodide; Rh: rhesus; TRITC: tetramethyl rhodamine isothiocyanate.

## Authors' contributions

JHK designed and made pDXA-mcs-RFPmars-hygro, created the fusion strains, designed and implemented all localization and protease experiments, prepared all final figures, and drafted the portions of this article unrelated to the ammonia assays. YX constructed the fusion protein plasmids and participated in creating the fusion strains. CTD participated in developing the methods for the colocalization and protease experiments. CKS conceived, designed and performed the ammonia transport assays, prepared the initial ammonia assay figures and drafted the ammonia assay portions of this article. JHK and CKS contributed equally in revising of the final manuscript.
